# *N*,*N*′-Di-*tert*-butyl-*N*′′-(2,6-difluoro­benzo­yl)phospho­ric triamide

**DOI:** 10.1107/S1600536810045927

**Published:** 2010-11-13

**Authors:** Mehrdad Pourayoubi, Atekeh Tarahhomi, Arnold L. Rheingold, James A. Golen

**Affiliations:** a Department of Chemistry, Ferdowsi University of Mashhad, Mashhad, 91779, Iran; b Department of Chemistry, University of California, San Diego, 9500 Gilman, Drive, La Jolla, CA 92093, USA

## Abstract

In the title compound, C_15_H_24_F_2_N_3_O_2_P, the phosphoryl and carbonyl groups adopt *anti* positions relative to each other. The P atom is in a tetra­hedral coordination environment and the environment of each N atom is essentially planar. In the crystal, adjacent mol­ecules are linked *via* N—H⋯O=P and N—H⋯O=C hydrogen bonds into an extended chain parallel to the *a* axis. The crystal studied was a non-merohedral twin with a minor twin component of 36.4 (1)%.

## Related literature

Carbacyl­amido­phosphates with a C(O)NHP(O) skeleton have attracted attention because of their roles as *O*,*O′*-donor ligands for metal complexation, see: Gholivand *et al.* (2010 ▶). *CELL_NOW* (Sheldrick, 2008*a* ▶) was used to generate the components of the twin.
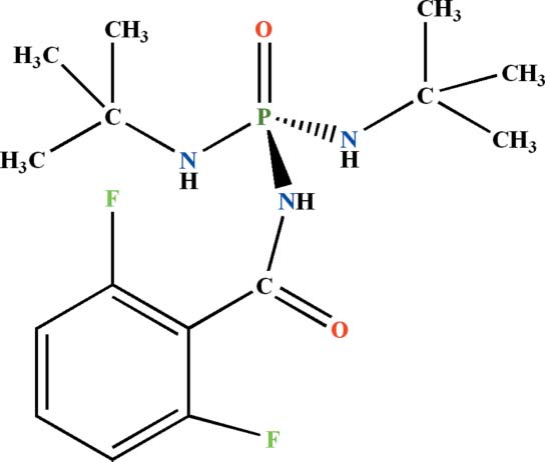


## Experimental

### 

#### Crystal data

C_15_H_24_F_2_N_3_O_2_P
*M* *_r_* = 347.34
Triclinic, 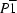

*a* = 9.8142 (12) Å
*b* = 10.2886 (13) Å
*c* = 10.6091 (16) Å
α = 117.171 (4)°
β = 98.636 (4)°
γ = 97.988 (3)°
*V* = 915.6 (2) Å^3^
*Z* = 2
Mo *K*α radiation
μ = 0.18 mm^−1^
*T* = 200 K
0.30 × 0.25 × 0.20 mm

#### Data collection

Bruker SMART X2S benchtop CCD area-detector diffractometer
Absorption correction: multi-scan (*TWINABS*; Sheldrick, 2008*a* ▶) *T*_min_ = 0.948, *T*_max_ = 0.965
7847 measured reflections
4225 independent reflections
3525 reflections with *I* > 2σ(*I*)
*R*_int_ = 0.057

#### Refinement

*R*[*F* ^2^ > 2σ(*F* ^2^)] = 0.049
*wR*(*F* ^2^) = 0.121
*S* = 1.05
4225 reflections
224 parameters
3 restraints
H atoms treated by a mixture of independent and constrained refinement
Δρ_max_ = 0.35 e Å^−3^
Δρ_min_ = −0.27 e Å^−3^

### 

Data collection: *GIS* (Bruker, 2009 ▶); cell refinement: *SAINT* (Bruker, 2009 ▶); data reduction: *SAINT*; program(s) used to solve structure: *SHELXS97* (Sheldrick, 2008*b* ▶); program(s) used to refine structure: *SHELXL97* (Sheldrick, 2008*b*); molecular graphics: *SHELXTL* (Sheldrick, 2008*b*); software used to prepare material for publication: *SHELXTL*.

## Supplementary Material

Crystal structure: contains datablocks I, global. DOI: 10.1107/S1600536810045927/ng5051sup1.cif

Structure factors: contains datablocks I. DOI: 10.1107/S1600536810045927/ng5051Isup2.hkl

Additional supplementary materials:  crystallographic information; 3D view; checkCIF report

## Figures and Tables

**Table 1:** Hydrogen-bond geometry (Å, °)

*D*—H⋯*A*	*D*—H	H⋯*A*	*D*⋯*A*	*D*—H⋯*A*
N1—H1*N*⋯O2^i^	0.86 (1)	1.96 (1)	2.808 (2)	172 (2)
N2—H2*N*⋯O1^ii^	0.86 (1)	2.22 (1)	3.042 (2)	160 (2)
N3—H3*N*⋯O1^ii^	0.86 (1)	2.22 (2)	3.008 (2)	152 (2)

Symmetry codes: (i) 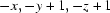; (ii) 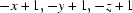.

**Fig. 1.**
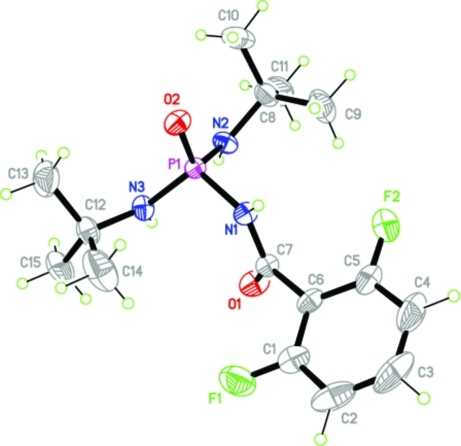
An ORTEP-style plot of title compound. Ellipsoids are given at the 50% probability level.

## supplementary crystallographic information

### Comment

Carbacylamidophosphates with a C(O)NHP(O) skeleton have attracted attention because of their roles as the *O*,*O'*-donor ligands for metal complexation (Gholivand *et al.*, 2010).

Here, we report on the synthesis and crystal structure of title carbacylamidophosphate, P(O)[NHC(O)C_6_H_3_(2,6-F_2_)][NHC(CH_3_)_3_]_2_. The phosphoryl and carbonyl groups adopt the *anti* position to each other. The P atom has a slightly distorted tetrahedral configuration (Fig. 1). The bond angles around the P atom are in the range of 102.21 (9)° to 116.57 (10)°. The P1–N2 and P1–N3 bonds (1.631 (2) Å and 1.6301 (18) Å) are shorter than the P1–N1 bond (1.7142 (17) Å). The environment of the nitrogen atoms is essentially planar. The P═O bond length of 1.4761 (16) Å is standard for phosphoramidate compounds.

In the crystal structure, adjacent molecules are linked *via* N–H···O═ P and N–H···O═C hydrogen bonds, into an extended chain parallel to the *a* axis. The crystals were found to be twinned.

### Experimental

2,6-F_2_—C_6_H_3_C(O)NHP(O)Cl_2_ has been synthesized from the reaction between phosphorus pentachloride (3.478 g, 16.7 mmol) and 2,6-difluorobenzamide (2.624 g, 16.7 mmol) in dry CCl_4_ at 358 K (3 h) and then the treatment of formic acid (0.769 g, 16.7 mmol) at ice bath temperature.

To a solution of 2,6-F_2_—C_6_H_3_C(O)NHP(O)Cl_2_ (0.500 g, 1.825 mmol) in dry CHCl_3_, a solution of *tert*-butylamine (0.534 g, 7.300 mmol) in dry CHCl_3_ (1:4 mole ratio) was added dropwise at 273 K. After 4 h of stirring, the solvent was evaporated at room temperature. The solid was washed with distilled water. Single crystals were obtained from a solution of the title compound in DMF/CH_3_OH and n-C_7_H_16_ after a slow evaporation at room temperature. Colorless crystal of the title compound was mounted on a Mitogen mount with epoxy and data was collected at 200 K on a Bruker *SMART* X2S system with Mo Kα radiation. IR (KBr, cm^-1^): 3351 (NH), 3094 (NH), 2960, 2202, 1665 (C═O), 1474, 1398, 1239 (P═O), 1020, 878 (*P*—N_amine_), 779 (*P*—N_amide_).

### Refinement

Structure was solved by direct methods and all non-hydrogen atoms were refined as being anisotropic by Fourier full matrix least squares on F^2^. Hydrogen atoms on various N atoms were found from a Fourier difference map and these N–H distances were then refined with the distance restraint N–H 0.87 (1) angstrom and with Uiso(H) = 1.2 Ueq(N). All other H atoms were placed in geometrically idealized positions and constrained to ride on their parent atoms with aromatic CH distances of 0.95 Å, Uiso(H) = 1.2Ueq(C) and with methyl C–H distances of 0.98 Å, Uiso(H) = 1.5Ueq(C).

Number of reflections and value of Rint were changed to indicate values given in .ABS, .PRP, and .LST files.

Plat 242 ALERT C - comment on C12 low Ueq in comparison to neighbors. C12 is central atom of a *tert*-butyl group and attached C atoms have higher Ueq values.

### Crystal data

**Table:**

C_15_H_24_F_2_N_3_O_2_P	*Z* = 2
*M**_r_* = 347.34	*F*(000) = 368
Triclinic, *P*1	*D*_x_ = 1.260 Mg m^−^^3^
*a* = 9.8142 (12) Å	Mo *K*α radiation, λ = 0.71073 Å
*b* = 10.2886 (13) Å	Cell parameters from 2837 reflections
*c* = 10.6091 (16) Å	θ = 2.2–27.9°
α = 117.171 (4)°	µ = 0.18 mm^−^^1^
β = 98.636 (4)°	*T* = 200 K
γ = 97.988 (3)°	Block, colorless
*V* = 915.6 (2) Å^3^	0.30 × 0.25 × 0.20 mm

### Data collection

**Table:**

Bruker SMART X2S benchtop CCD area-detector diffractometer	4225 independent reflections
Radiation source: micro focus sealed tube	3525 reflections with *I* > 2σ(*I*)
doubly curved silicon crystal	*R*_int_ = 0.057
φ and ω scans	θ_max_ = 27.9°, θ_min_ = 2.2°
Absorption correction: multi-scan (*TWINABS*; Sheldrick, 2008*a*)	*h* = −12→12
*T*_min_ = 0.948, *T*_max_ = 0.965	*k* = −13→12
7847 measured reflections	*l* = 0→13

### Refinement

**Table:**

Refinement on *F*^2^	Primary atom site location: structure-invariant direct methods
Least-squares matrix: full	Secondary atom site location: difference Fourier map
*R*[*F*^2^ > 2σ(*F*^2^)] = 0.049	Hydrogen site location: inferred from neighbouring sites
*wR*(*F*^2^) = 0.121	H atoms treated by a mixture of independent and constrained refinement
*S* = 1.05	*w* = 1/[σ^2^(*F*_o_^2^) + (0.0548*P*)^2^ + 0.3384*P*] where *P* = (*F*_o_^2^ + 2*F*_c_^2^)/3
4225 reflections	(Δ/σ)_max_ = 0.007
224 parameters	Δρ_max_ = 0.35 e Å^−^^3^
3 restraints	Δρ_min_ = −0.26 e Å^−^^3^

### Special details

**Table:**

Experimental. Data refinement indicated a twin system and program Cell_Now (Sheldrick, 2008) was used to generate the two components of the twin (63.6 (1)/36.4 ratio). Data was integrated using *SAINT* and corrected for absorption using TWINABS (Shelrick, 2008).
Geometry. All e.s.d.'s (except the e.s.d. in the dihedral angle between two l.s. planes) are estimated using the full covariance matrix. The cell e.s.d.'s are taken into account individually in the estimation of e.s.d.'s in distances, angles and torsion angles; correlations between e.s.d.'s in cell parameters are only used when they are defined by crystal symmetry. An approximate (isotropic) treatment of cell e.s.d.'s is used for estimating e.s.d.'s involving l.s. planes.
Refinement. Refinement of *F*^2^ against ALL reflections. The weighted *R*-factor *wR* and goodness of fit *S* are based on *F*^2^, conventional *R*-factors *R* are based on *F*, with *F* set to zero for negative *F*^2^. The threshold expression of *F*^2^ > σ(*F*^2^) is used only for calculating *R*-factors(gt) *etc*. and is not relevant to the choice of reflections for refinement. *R*-factors based on *F*^2^ are statistically about twice as large as those based on *F*, and *R*- factors based on ALL data will be even larger.

### Fractional atomic coordinates and isotropic or equivalent isotropic displacement parameters (Å^2^)

**Table:**

	*x*	*y*	*z*	*U*_iso_*/*U*_eq_	
P1	0.18621 (6)	0.39181 (6)	0.41180 (6)	0.02191 (14)	
F1	0.4134 (2)	0.6847 (2)	0.94162 (17)	0.0611 (5)	
F2	0.23506 (19)	0.89470 (17)	0.66107 (18)	0.0523 (4)	
O1	0.45094 (16)	0.62402 (19)	0.64132 (18)	0.0363 (4)	
O2	0.03152 (16)	0.32970 (16)	0.36977 (17)	0.0305 (4)	
N1	0.20980 (18)	0.55903 (19)	0.56848 (19)	0.0241 (4)	
H1N	0.1375 (17)	0.592 (3)	0.595 (3)	0.029*	
N2	0.2528 (2)	0.4227 (2)	0.29337 (19)	0.0276 (4)	
H2N	0.3378 (14)	0.411 (3)	0.293 (3)	0.033*	
N3	0.28600 (19)	0.2910 (2)	0.4441 (2)	0.0260 (4)	
H3N	0.3739 (13)	0.318 (3)	0.449 (3)	0.031*	
C1	0.3611 (3)	0.7959 (3)	0.9332 (3)	0.0404 (6)	
C2	0.3480 (4)	0.9159 (4)	1.0587 (3)	0.0597 (9)	
H2A	0.3767	0.9216	1.1511	0.072*	
C3	0.2927 (4)	1.0271 (4)	1.0475 (4)	0.0675 (10)	
H3A	0.2807	1.1087	1.1327	0.081*	
C4	0.2544 (3)	1.0216 (3)	0.9142 (4)	0.0593 (9)	
H4A	0.2183	1.0995	0.9069	0.071*	
C5	0.2699 (3)	0.8998 (3)	0.7919 (3)	0.0386 (6)	
C6	0.3228 (2)	0.7840 (2)	0.7961 (2)	0.0283 (5)	
C7	0.3352 (2)	0.6488 (2)	0.6613 (2)	0.0243 (4)	
C8	0.1874 (3)	0.4811 (3)	0.1991 (2)	0.0353 (5)	
C9	0.1621 (4)	0.6356 (3)	0.2928 (3)	0.0580 (9)	
H9A	0.2518	0.7042	0.3598	0.087*	
H9B	0.0940	0.6275	0.3491	0.087*	
H9C	0.1241	0.6747	0.2297	0.087*	
C10	0.0477 (3)	0.3691 (4)	0.0967 (3)	0.0569 (8)	
H10A	−0.0227	0.3709	0.1536	0.085*	
H10B	0.0640	0.2677	0.0478	0.085*	
H10C	0.0126	0.3972	0.0234	0.085*	
C11	0.2913 (4)	0.4907 (4)	0.1095 (3)	0.0535 (7)	
H11A	0.3809	0.5611	0.1750	0.080*	
H11B	0.2515	0.5263	0.0439	0.080*	
H11C	0.3084	0.3911	0.0518	0.080*	
C12	0.2566 (3)	0.1929 (3)	0.5095 (3)	0.0336 (5)	
C13	0.1325 (3)	0.0588 (3)	0.4049 (4)	0.0579 (8)	
H13A	0.1533	0.0073	0.3088	0.087*	
H13B	0.0460	0.0945	0.3956	0.087*	
H13C	0.1188	−0.0112	0.4433	0.087*	
C14	0.2251 (5)	0.2791 (4)	0.6581 (4)	0.0677 (10)	
H14A	0.3057	0.3642	0.7232	0.102*	
H14B	0.2093	0.2123	0.6997	0.102*	
H14C	0.1400	0.3165	0.6470	0.102*	
C15	0.3893 (3)	0.1343 (4)	0.5243 (4)	0.0525 (7)	
H15A	0.4090	0.0802	0.4280	0.079*	
H15B	0.3742	0.0661	0.5645	0.079*	
H15C	0.4701	0.2191	0.5898	0.079*	

### Atomic displacement parameters (Å^2^)

**Table:**

	*U*^11^	*U*^22^	*U*^33^	*U*^12^	*U*^13^	*U*^23^
P1	0.0176 (2)	0.0218 (2)	0.0242 (3)	0.00825 (19)	0.00585 (19)	0.0082 (2)
F1	0.0786 (14)	0.0696 (11)	0.0426 (9)	0.0253 (10)	0.0115 (8)	0.0322 (9)
F2	0.0626 (11)	0.0417 (8)	0.0565 (10)	0.0198 (8)	0.0103 (8)	0.0264 (8)
O1	0.0199 (7)	0.0431 (9)	0.0381 (9)	0.0093 (7)	0.0107 (7)	0.0117 (7)
O2	0.0185 (7)	0.0288 (7)	0.0359 (8)	0.0083 (6)	0.0062 (6)	0.0083 (7)
N1	0.0179 (8)	0.0251 (8)	0.0258 (8)	0.0100 (7)	0.0080 (7)	0.0073 (7)
N2	0.0238 (9)	0.0369 (10)	0.0278 (9)	0.0147 (8)	0.0093 (8)	0.0174 (8)
N3	0.0190 (9)	0.0280 (9)	0.0367 (10)	0.0111 (7)	0.0116 (8)	0.0173 (8)
C1	0.0370 (14)	0.0419 (13)	0.0338 (12)	0.0027 (11)	0.0089 (10)	0.0131 (11)
C2	0.0572 (19)	0.067 (2)	0.0259 (13)	0.0018 (16)	0.0120 (12)	0.0022 (13)
C3	0.061 (2)	0.0446 (17)	0.0517 (18)	0.0063 (15)	0.0210 (16)	−0.0140 (14)
C4	0.0560 (19)	0.0317 (13)	0.067 (2)	0.0160 (13)	0.0164 (16)	0.0030 (14)
C5	0.0337 (13)	0.0274 (11)	0.0418 (13)	0.0036 (10)	0.0092 (11)	0.0072 (10)
C6	0.0205 (9)	0.0265 (10)	0.0276 (10)	0.0022 (8)	0.0075 (8)	0.0052 (8)
C7	0.0210 (10)	0.0264 (10)	0.0252 (10)	0.0067 (8)	0.0083 (8)	0.0112 (8)
C8	0.0437 (14)	0.0373 (12)	0.0308 (11)	0.0163 (11)	0.0077 (10)	0.0198 (10)
C9	0.087 (3)	0.0501 (16)	0.0516 (16)	0.0393 (17)	0.0195 (16)	0.0298 (14)
C10	0.0501 (18)	0.0689 (19)	0.0498 (16)	0.0046 (14)	−0.0106 (13)	0.0366 (15)
C11	0.068 (2)	0.0642 (18)	0.0426 (15)	0.0200 (16)	0.0189 (14)	0.0346 (14)
C12	0.0338 (13)	0.0360 (12)	0.0445 (13)	0.0173 (10)	0.0172 (10)	0.0259 (11)
C13	0.0462 (18)	0.0477 (16)	0.087 (2)	0.0019 (13)	0.0107 (16)	0.0424 (17)
C14	0.108 (3)	0.075 (2)	0.0601 (19)	0.055 (2)	0.052 (2)	0.0477 (18)
C15	0.0503 (17)	0.0584 (17)	0.0721 (19)	0.0294 (14)	0.0168 (15)	0.0456 (16)

### Geometric parameters (Å, °)

**Table:**

P1—O2	1.4761 (16)	C8—C9	1.522 (4)
P1—N3	1.6301 (18)	C8—C10	1.536 (4)
P1—N2	1.631 (2)	C9—H9A	0.9800
P1—N1	1.7142 (17)	C9—H9B	0.9800
F1—C1	1.351 (3)	C9—H9C	0.9800
F2—C5	1.353 (3)	C10—H10A	0.9800
O1—C7	1.226 (2)	C10—H10B	0.9800
N1—C7	1.352 (3)	C10—H10C	0.9800
N1—H1N	0.858 (10)	C11—H11A	0.9800
N2—C8	1.495 (3)	C11—H11B	0.9800
N2—H2N	0.860 (10)	C11—H11C	0.9800
N3—C12	1.485 (3)	C12—C14	1.520 (4)
N3—H3N	0.856 (10)	C12—C15	1.526 (3)
C1—C2	1.381 (4)	C12—C13	1.532 (4)
C1—C6	1.390 (4)	C13—H13A	0.9800
C2—C3	1.377 (5)	C13—H13B	0.9800
C2—H2A	0.9500	C13—H13C	0.9800
C3—C4	1.381 (6)	C14—H14A	0.9800
C3—H3A	0.9500	C14—H14B	0.9800
C4—C5	1.380 (4)	C14—H14C	0.9800
C4—H4A	0.9500	C15—H15A	0.9800
C5—C6	1.381 (3)	C15—H15B	0.9800
C6—C7	1.509 (3)	C15—H15C	0.9800
C8—C11	1.517 (4)		
			
O2—P1—N3	116.57 (10)	C8—C9—H9A	109.5
O2—P1—N2	115.98 (9)	C8—C9—H9B	109.5
N3—P1—N2	102.21 (9)	H9A—C9—H9B	109.5
O2—P1—N1	103.22 (8)	C8—C9—H9C	109.5
N3—P1—N1	109.37 (9)	H9A—C9—H9C	109.5
N2—P1—N1	109.43 (10)	H9B—C9—H9C	109.5
C7—N1—P1	126.27 (14)	C8—C10—H10A	109.5
C7—N1—H1N	113.8 (16)	C8—C10—H10B	109.5
P1—N1—H1N	119.9 (16)	H10A—C10—H10B	109.5
C8—N2—P1	127.01 (16)	C8—C10—H10C	109.5
C8—N2—H2N	118.1 (19)	H10A—C10—H10C	109.5
P1—N2—H2N	114.6 (19)	H10B—C10—H10C	109.5
C12—N3—P1	128.21 (15)	C8—C11—H11A	109.5
C12—N3—H3N	114.1 (19)	C8—C11—H11B	109.5
P1—N3—H3N	114.8 (18)	H11A—C11—H11B	109.5
F1—C1—C2	119.7 (3)	C8—C11—H11C	109.5
F1—C1—C6	117.6 (2)	H11A—C11—H11C	109.5
C2—C1—C6	122.7 (3)	H11B—C11—H11C	109.5
C3—C2—C1	118.8 (3)	N3—C12—C14	111.4 (2)
C3—C2—H2A	120.6	N3—C12—C15	106.2 (2)
C1—C2—H2A	120.6	C14—C12—C15	110.4 (2)
C2—C3—C4	120.9 (3)	N3—C12—C13	109.3 (2)
C2—C3—H3A	119.5	C14—C12—C13	110.8 (3)
C4—C3—H3A	119.5	C15—C12—C13	108.5 (2)
C3—C4—C5	118.3 (3)	C12—C13—H13A	109.5
C3—C4—H4A	120.9	C12—C13—H13B	109.5
C5—C4—H4A	120.9	H13A—C13—H13B	109.5
F2—C5—C4	118.7 (3)	C12—C13—H13C	109.5
F2—C5—C6	117.9 (2)	H13A—C13—H13C	109.5
C4—C5—C6	123.4 (3)	H13B—C13—H13C	109.5
C5—C6—C1	116.0 (2)	C12—C14—H14A	109.5
C5—C6—C7	123.1 (2)	C12—C14—H14B	109.5
C1—C6—C7	120.9 (2)	H14A—C14—H14B	109.5
O1—C7—N1	124.00 (18)	C12—C14—H14C	109.5
O1—C7—C6	121.48 (18)	H14A—C14—H14C	109.5
N1—C7—C6	114.52 (17)	H14B—C14—H14C	109.5
N2—C8—C11	106.6 (2)	C12—C15—H15A	109.5
N2—C8—C9	110.46 (19)	C12—C15—H15B	109.5
C11—C8—C9	110.4 (2)	H15A—C15—H15B	109.5
N2—C8—C10	109.0 (2)	C12—C15—H15C	109.5
C11—C8—C10	109.5 (2)	H15A—C15—H15C	109.5
C9—C8—C10	110.8 (2)	H15B—C15—H15C	109.5
			
O2—P1—N1—C7	169.82 (18)	C4—C5—C6—C7	178.0 (2)
N3—P1—N1—C7	45.1 (2)	F1—C1—C6—C5	−180.0 (2)
N2—P1—N1—C7	−66.1 (2)	C2—C1—C6—C5	−0.3 (4)
O2—P1—N2—C8	38.0 (2)	F1—C1—C6—C7	2.0 (3)
N3—P1—N2—C8	165.93 (18)	C2—C1—C6—C7	−178.3 (2)
N1—P1—N2—C8	−78.2 (2)	P1—N1—C7—O1	3.1 (3)
O2—P1—N3—C12	−33.9 (2)	P1—N1—C7—C6	−175.92 (16)
N2—P1—N3—C12	−161.48 (19)	C5—C6—C7—O1	114.8 (3)
N1—P1—N3—C12	82.6 (2)	C1—C6—C7—O1	−67.3 (3)
F1—C1—C2—C3	−179.2 (3)	C5—C6—C7—N1	−66.1 (3)
C6—C1—C2—C3	1.1 (5)	C1—C6—C7—N1	111.8 (2)
C1—C2—C3—C4	−1.8 (5)	P1—N2—C8—C11	179.62 (18)
C2—C3—C4—C5	1.5 (5)	P1—N2—C8—C9	59.7 (3)
C3—C4—C5—F2	−178.8 (3)	P1—N2—C8—C10	−62.3 (3)
C3—C4—C5—C6	−0.6 (4)	P1—N3—C12—C14	−55.9 (3)
F2—C5—C6—C1	178.2 (2)	P1—N3—C12—C15	−176.14 (19)
C4—C5—C6—C1	0.0 (4)	P1—N3—C12—C13	67.0 (3)
F2—C5—C6—C7	−3.8 (3)		

### Hydrogen-bond geometry (Å, °)

**Table:**

*D*—H···*A*	*D*—H	H···*A*	*D*···*A*	*D*—H···*A*
N1—H1N···O2^i^	0.86 (1)	1.96 (1)	2.808 (2)	172 (2)
N2—H2N···O1^ii^	0.86 (1)	2.22 (1)	3.042 (2)	160 (2)
N3—H3N···O1^ii^	0.86 (1)	2.22 (2)	3.008 (2)	152 (2)

Symmetry codes: (i) −*x*, −*y*+1, −*z*+1; (ii) −*x*+1, −*y*+1, −*z*+1.
